# Gαi2^+^ vomeronasal neurons govern the initial outcome of an acute social competition

**DOI:** 10.1038/s41598-020-57765-6

**Published:** 2020-01-21

**Authors:** Anna Pallé, Marta Montero, Silvia Fernández, Patricia Tezanos, Juan A. de las Heras, Valerie Luskey, Lutz Birnbaumer, Frank Zufall, Pablo Chamero, José Luis Trejo

**Affiliations:** 1 Anna Pallé. Cajal Institute, Dept Translational Neuroscience, CSIC, Madrid, 28002 Spain; 2 Marta Montero. Cajal Institute, Dept Translational Neuroscience, CSIC, Madrid, 28002 Spain; 3 Silvia Fernandez. Cajal Institute, Molecular and Cellular Biology Unit, CSIC, Madrid, 28002 Spain; 4 Patricia Tezanos. Cajal Institute, Dept Translational Neuroscience, CSIC, Madrid, 28002 Spain; 5 Juan A. de las Heras. Cajal Institute, Dept Translational Neuroscience, CSIC, Madrid, 28002 Spain; 6 Valerie Luskey. Cajal Institute, Dept Translational Neuroscience, CSIC, Madrid, 28002 Spain; 7 Lutz Birnbaumer. Neurobiology Laboratory, National Institute of Environmental Health Sciences, National Institutes of Health, Durham, NC 27709 USA; 8 Institute of Biomedical Research (BIOMED), School of Medical Sciences, Catholic University of Argentina, Buenos Aires, C1107AAZ Argentina; 9 Frank Zufall. Center for Integrative Physiology and Molecular Medicine, Saarland University, 66421 Homburg, Germany; 10 Pablo Chamero. Laboratoire de Physiologie de la Reproduction et des Comportements, UMR 0085 INRAE-CNRS-IFCE-University of Tours, Nouzilly, 37380 France; 11 José Luis Trejo. Cajal Institute, Dept Translational Neuroscience, CSIC, Madrid, 28002 Spain

**Keywords:** Social behaviour, Olfactory bulb

## Abstract

Pheromone detection by the vomeronasal organ (VNO) mediates important social behaviors across different species, including aggression and sexual behavior. However, the relationship between vomeronasal function and social hierarchy has not been analyzed reliably. We evaluated the role of pheromone detection by receptors expressed in the apical layer of the VNO such as vomeronasal type 1 receptors (V1R) in dominance behavior by using a conditional knockout mouse for G protein subunit Gαi2, which is essential for V1R signaling. We used the tube test as a model to analyze the within-a-cage hierarchy in male mice, but also as a paradigm of novel territorial competition in animals from different cages. In absence of prior social experience, Gαi2 deletion promotes winning a novel social competition with an unfamiliar control mouse but had no effect on an established hierarchy in cages with mixed genotypes, both Gαi2^−/−^ and controls. To further dissect social behavior of Gαi2^−/−^ mice, we performed a 3-chamber sociability assay and found that mutants had a slightly altered social investigation. Finally, gene expression analysis in the medial prefrontal cortex (mPFC) for a subset of genes previously linked to social status revealed no differences between group-housed Gαi2^−/−^ and controls. Our results reveal a direct influence of pheromone detection on territorial dominance, indicating that olfactory communication involving apical VNO receptors like V1R is important for the outcome of an initial social competition between two unfamiliar male mice, whereas final social status acquired within a cage remains unaffected. These results support the idea that previous social context is relevant for the development of social hierarchy of a group. Overall, our data identify two context-dependent forms of dominance, acute and chronic, and that pheromone signaling through V1R receptors is involved in the first stages of a social competition but in the long term is not predictive for high social ranks on a hierarchy.

## Introduction

Social dominance comprises a set of behaviors related to the control of resources between animals of the same species, including access to territory, reproduction, and food^1^. Such imposition can be either chronic or acute when animals face either repetitive or sporadic encounters. In group-living animals, repetitive encounters of animals living in the same group can lead to social dominance, i.e. social hierarchy, conditioned by the group’s social history^2^. In non-group living animals, territorial control can occur as a result of acute dyadic interactions^3^ between animals with no record of previous contact. Whether these two different types of dominance are regulated by the same neural mechanisms is currently unclear.

The mechanisms that may mediate social dominance are likely to involve the sensing of pheromones, which in mammals largely depends on the vomeronasal organ (VNO), a chemosensory organ with a crucial role in mediating different innate behaviors^4–6^ including intermale aggression^7^. Some studies postulate that aggression is needed for the display of dominance behaviors^8,9^. Intermale aggression, through optogenetic stimulation of PMv^DAT^ (dopamine transporter-expressing neurons in the hypothalamic ventral premammillary nucleus), shifted the dominance status in pairs of mice, suggesting that the brain networks involved in aggressive behavior may also be implicated in dominance^10^. However, a number of reports failed to find a direct correlation between aggression and dominance, suggesting that aggression is not always necessary to establish a social dominance hierarchy, or that such correlation could exist only in certain species^11^ such as fishes^12^, and laboratory mice^13^. Thus, although aggression can be used to initially generate a hierarchy, once stable, it may not be exclusive of dominant individuals and rather depend upon social context^14^. Thus, it is currently not known whether the detection of pheromones by the VNO may have a role in some types of social dominance (for example, whether pheromone signaling-deficient mice are more dominant than controls), and whether this influence on dominance can be analyzed independently of aggression. The tube test has recently emerged as a reliable method for measuring territorial dominance through dyadic interactions, and we have used this test along with a round-robin design. Classical methods of measuring dominance typically rely on ethological measures of agonistic interactions within the cage, or number of attacks and submissive retreats after an aggressive interaction^14^. However, this observational analysis for measuring hierarchy is limited by the bias that relies on the assumption that dominants display more frequently aggressive attacks than subordinate individuals. In contrast, tube test and round-robin design data correlate more reliably with the social rank measured in other tests, like the visible burrow system, measures of ultrasonic vocalizations, or urine marking patterns^15,16^, without relying on measures of aggressiveness. Therefore, the tube test emerges as a useful tool to address the relationship between dominance and aggression.

Pheromones are detected in the VNO by different subsets of vomeronasal sensory neurons (VSNs) defined by the expression of V1R and V2R G protein-coupled receptors^17^. Each subset of V1R and V2R-expressing VSNs leads male-male aggressive behavior in opposite directions: mutant mice for Gαo in which sensory signal transduction mediated by V2Rs is inactivated, do not display territorial aggression in the resident-intruder paradigm^17,18^, while ablation of Gαi2 increases territorial aggression^19^. Whether disruption of V1R pheromone detection and subsequent increase in male-male aggression have an influence in the establishment of social dominance remains unexplored. Further, we recently showed overexpression of a V1R gene and some olfactory receptor genes in the mPFC of dominant animals compared to subordinates^20^. These findings suggest a potential link between social hierarchy and vomeronasal function.

Here, we used the tube test in mice with Gαi2 deficiency in vomeronasal neurons to analyze the role of pheromone detection by the VNO in social dominance, measured either as first-time encounters by pairs of mice, or as group-living animals’ social hierarchy. We also measured social preference to analyze whether putative differences in sociability could account for the differences between both types of dominance. Finally, we compared the expression of a number of olfactory and vomeronasal receptor genes between Gαi2-deficient animals and control littermates in the medial prefrontal cortex (mPFC) by qPCR. Together, our results suggest an unexpected neural mechanism that integrates vomeronasal function and pheromone detection with chronic and acute social dominance and social investigation.

## Results

### Gαi2-KO mutant mice form stable and linear social hierarchies

**Figure 1:**
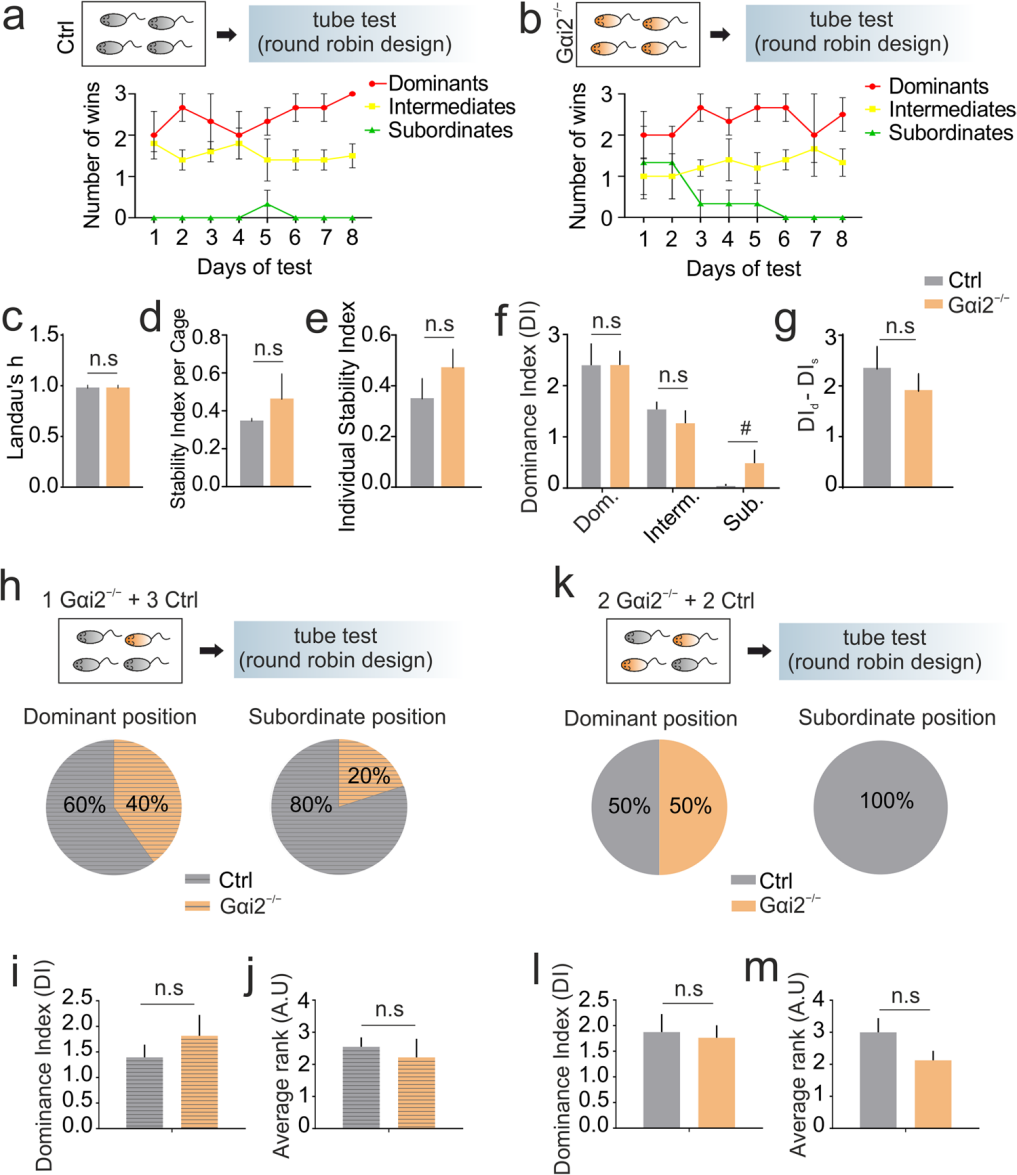
Gαi2 deficiency in the vomeronasal organ does not affect hierarchy stability or dominance behavior in group-housed animals. (**a**–**g**) Single genotype per cage, either control or Gαi2^−/−^ mice. (**h**–**m**) Mixed genotypes per cage, either one Gαi2^−/−^ plus three controls, or two Gαi2^−/−^ plus two controls per cage. (**a**,**b**) Hierarchy rank time course in the tube test measured by the number of winnings per animal per day ((**a**) group-housed controls; (**b**) group-housed Gαi2^−/−^ mice). (**c**–**e**) Properties of within-a-cage hierarchy. (**c**) Linearity of dominance measured by Landau’s h index, Mann-Whitney U test, (p = 1) revealed no differences, n = 3 cages per group. (**d**) Stability per cage, Mann-Whitney U test, (p = 0.507) revealed no differences, n = 3 cages per group. (**e**) Stability per individual, Mann-Whitney U test (p = 0.242) revealed no differences between groups, n = 11 per group. (**f**) Dominance index (DI) for each rank. T-test (dominants p = 0.412 and intermediates p = 0.382) revealed no differences between groups for each rank. In subordinates, a trend to higher DI in Gαi2^−/−^ was observed. (Mann-Whitney U test p = 0.072). (**g**) DI difference between highest- and lowest-ranked animals into each cage. Mann-Whitney U test revealed no differences between genotypes, (p = 0.275), n = 3 per group. (**h**) Genotype frequency per hierarchy ranks (dominant and subordinate positions), Chi-square for % dominants (Chi = 0.8, p = 0.371) and for % of subordinates (Chi = 0.089, p = 0.766). (**i**) Mean DI per genotype, Mann-Whitney U test (p = 0.275), revealed no differences between controls and Gαi2^−/−^ mice, n = 15 controls, n = 5 KOs. (**j**) Average hierarchy rank per genotype, Mann-Whitney U test (p = 0.558) revealed no influence of genotype on the final social rank, n = 15 controls, n = 5 KOs. (**k**) Genotype frequency per hierarchy ranks (dominant and subordinate positions), Chi-square for % of dominants (Chi: 0.018, p = 0.893) and for % of subordinates (Chi: 6,296, p = 0.012). (**l**) Mean DI per genotype, T-test (p = 0.798), revealed no differences between controls and Gαi2^−/−^ mice, n = 10 per group. (**m**) Average hierarchy rank per genotype, T-test (p = 0.129) revealed no influence of genotype on the final social rank, n = 9 controls, n = 8 KOs. All data are shown as mean ± SEM. Inter-group effects are shown as: *p < 0.05, **p < 0.01, ***p < 0.001. Tendencies are represented as 0.05 > ^#^p < 0.1.

Conditional loss of Gαi2 in the olfactory system impairs sensory signaling in apical VSNs and abolishes neural response to V1R ligands^19^. To test whether the loss of Gαi2 could affect social hierarchy parameters, such as linearity, transitivity, and stability^16^, we group-housed Gαi2^−/−^ and control males (both Gαi2^+/−^ and wild-type) in different cages. After 4–6 weeks of cohabitation, mice of the same cage were tested in the tube test in a round-robin design to assess the hierarchy (Fig. 1a,b). Sociomatrices of win/loss were created in order to analyze linearity through the “Compete” package of R^21^. By calculating Landau’s h index, we confirmed that Gαi2^−/−^ are able to form hierarchies that have similar linearity compared to controls (Fig. 1c). To our knowledge, no previous mathematical index has been described to assess the stability of an existent hierarchy. To test whether the lack of Gαi2 affects the stability of an individual in the within-a-cage hierarchy ranking, we created two different stability coefficients. The Pallé-Luskey Stability Index (PLSI) calculates the average change of rank of each individual along the days of the test, assessing both individual stability for each animal but also the within-a-cage average stability of the group. The Tezanos Stability Index (TSI) evaluates the stability of a cage group by comparing the variance of the dominance index among individuals of each hierarchy to a hypothetically perfect stability cage group. PLSI and TSI formulas are shown in Methods section. We found neither differences in PLSI or TSI for the individual stability between Gαi2^−/−^ individuals compared to controls, nor in the average of cages from each genotype (Figs. 1d,e and S1). Cages from both genotypes showed clear differentiated social ranks between dominants and subordinates (Fig. 1f) although a trend was observed for a higher dominance index (DI) in subordinates from Gαi2^−/−^ cages. Importantly, no differences were found between genotypes with respect to their higher and lower dominance index (Fig. 1g), indicating that mutant mice display stable hierarchies.

We observed no differences in body weight (Fig. S2) and only some minor differences in the latency to cross the tube in the training phase in Gαi2^−/−^ mice (Fig. S3). The control group, comprising of wild-type and Gαi2^+/−^ mice behaved similarly in the parameters of indirect mobility analyzed (Fig. S3). Taken together, these results suggest that differences in the mobility within the tube and in weight are not accountable for the outcome of the matches in following experimental days. In conclusion, Gαi2 deficiency does not affect the innate ability to form stable hierarchies and does not affect basic physiological parameters potentially underlying dominance such as mobility or weight.

### Lack of Gαi2 does not affect the dominance index between mutants and controls in a social hierarchy test

Conditional deletion of Gαi2 in VSNs results in an increased number of attacks in the resident-intruder paradigm and causes increased activation in three distinct brain regions implicated in aggression^19^. We asked whether these changes could be associated with higher social rank. To address this, male mice were group-housed in cages consisting of one Gαi2^−/−^ and 3 controls, and we then calculated the social rank of these individuals using the tube test (Fig. 1h–j). We found that mutants were 40% of the total pool of dominants and 20% of the subordinates (Fig. 1h), which differs from what it would be expected from a random distribution (25% and 75%, respectively). However, no significant differences were found in the dominance index (Fig. 1i, see methods) or in the average rank position (Fig. 1j). Comparison of dominance indexes under these experimental conditions could be limited to the asymmetry of N size between Gαi2^−/−^ and controls. We therefore used a second experimental design in which Gαi2^−/−^ and controls were housed in a 2:2 ratio (Fig. 1k–m). Rank analysis revealed that both KOs and controls had an equal probability to belong to dominant positions, as expected for a random trial. However, none of the mutant mice belonged to subordinate positions (Fig. 1k). Nonetheless, we found no difference in the dominance index between KOs and controls (Fig. 1l), or in the average rank position of each group (Fig. 1m). To further analyze the nature of each confrontation between genotypes (time spent to win and time spent to push the other out of the tube), we scored the average latency to win a confrontation (Fig. S4a,b). We found no significant differences between groups. Together, these results indicate that Gαi2^−/−^ mice do not display a stronger dominance behavior, and that they can obtain any position within a given hierarchy from dominant to subordinate, but are frequently found in intermediate ranks.

### Gαi2 deletion in vomeronasal neurons promotes winning a novel social competition

**Figure 2:**
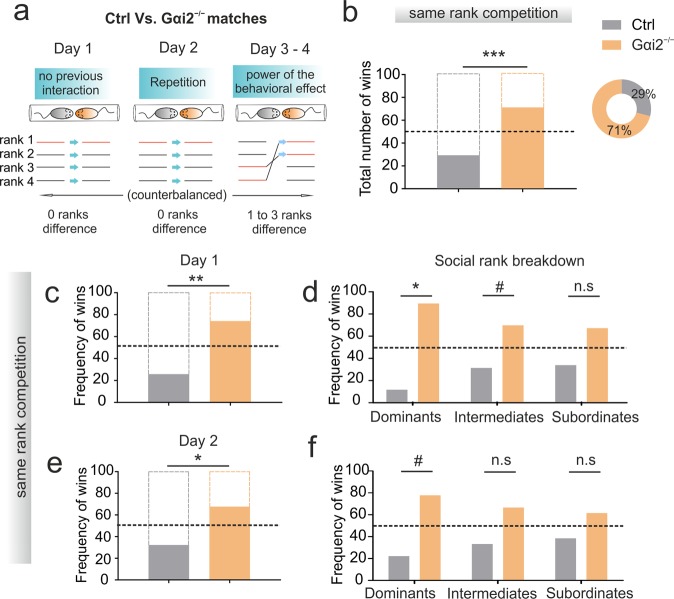
Disruption of the vomeronasal receptor type 1 signaling cascade in vomeronasal neurons enhances winning probability of a social competition with an unfamiliar male. (**a**) Encounters with unfamiliars in the tube test, experimental design. On day 1, animals with the same rank from different cages (different genotypes) were confronted in the tube test for the first time (both unfamiliar). Day 2 is a repetition of the same trials. The previous rank was obtained during the period of group-living with animals of the same genotype. On day 3 and 4, animals from different ranks were confronted. Pairs of animals from different genotype differing in their ranks by one position (day 3) or differing by three positions (day 4) were confronted. All comparisons were made to the random probability of winning (horizontal dot lines). (**b**) Total number and frequency of winnings on the first two days (day 1 and day 2), binomial distribution p = 0.0004 n = 62 trials; Gαi2^−/−^ mice won significantly higher than expected by chance, considering both days together or by measuring each day separately (**c**,**e**). (**c**) Total number of winnings on day 1, binomial distribution p = 0.00367 n = 31 trials. (**d**) Breakdown by hierarchy rank of the winning frequency on day 1, binomial distribution dominants (p = 0.01758) n = 9 trials, intermediates (0.08728) n = 13 trials, subordinates (p = 0.16406) n = 9 trials. In the dominant rank, Gαi2^−/−^ mice exhibited a significantly higher winning frequency both in the first and second day (**f**). In the intermediate rank, Gαi2^−/−^ mice outscored controls only the first day, while no differences were found in subordinates. (**e**) Total number of winnings on day 2, binomial distribution p = 0.00367 n = 31 trials, binomial distribution p = 0.02065. n = 31 trials. (**f**) Breakdown by hierarchy rank of the winnings frequency on day 1, binomial distribution dominants n = 9 (p = 0.07031), intermediates n = 13 (p = 0.1571), subordinates n = 9 trials (p = 0.16406). All data are shown as mean ± SEM. Inter-group effects are shown as: *p < 0.05, **p < 0.01, ***p < 0.001.

We next asked whether continuous social interactions within-a-cage may account for the former results. We tested the same genotype-grouped animals in a second phase using a paradigm of social competition in the absence of previous social hierarchy and cohabitation. Animals from different cages and genotypes that had never previously interacted were now confronted in the tube test for 4 consecutive days. During the first two days, KO vs. control animals of the same rank were confronted (Fig. 2a). We found that Gαi2^−/−^ animals showed a significantly higher frequency of wins than controls on both days (Fig. 2b). This effect was more robust the first day of the test, when animals had never had any previous interaction (Fig. 2c,e). Differences were more prevalent for higher ranks of the hierarchy, being significant for dominant mice and with a trend in intermediates but not in subordinates on the first day of the test, and only a trend for dominants the second one (Fig. 2d,f).

**Figure 3:**
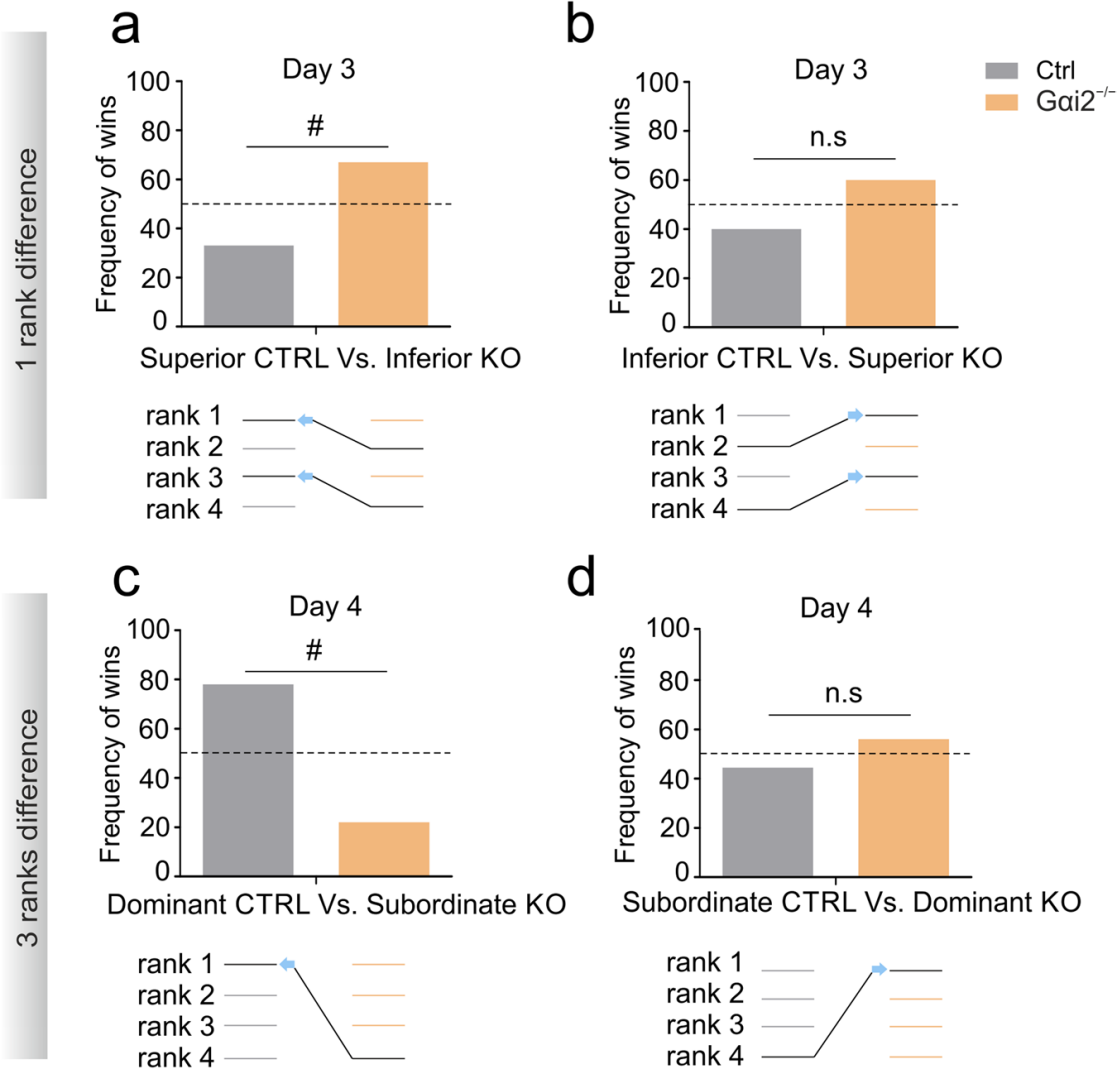
Increased winning probability during the first two days in Gαi2^−/−^ mice fades after test repetition (days 3 and 4). Tube test encounters between animals of different genotype and either one position difference (**a**,**b**) or three positions difference (**c**,**d**) in previous hierarchy ranks. (**a**) Winning frequency per genotype when superior ranked controls were paired with inferior ranked KO animals (rank 1 vs. rank 2, or rank 3 vs. rank 4). Gαi2^−/−^ mice presented a trend for increased winning frequency, Binomial distribution (p = 0.09164). This outcome was not significantly different when inferior-ranked controls were paired with higher-ranked KO animals (**b**), Binomial distribution (p = 0.15274). A similar trend to fade previous winning probabilities was found on day 4, when the highest possible rank differences were confronted (dominant vs. subordinates). (**c**) Dominant controls presented a trend for increased winning probability when paired with subordinate KOs, binomial distribution (p = 0.07). (**d**) No significant differences were found when subordinate controls were paired with dominant KOs, binomial distribution (p = 0.24609). All data are shown as mean ± SEM. Inter-group effects are shown as: *p < 0.05, **p < 0.01, ***p < 0.001.

We next matched controls vs KOs from different ranks to increase the difficulty of the test: one rank difference on day 3 (Fig. 3a,b) and 3 ranks difference on day 4 (Fig. 3c,d). On day 3, we found that more KO animals from inferior rank won confrontations when matched with superior control ranks (statistical trend; Fig. 3a). Differences between inferior-ranked controls matched against superior-ranked KOs were not significant (Fig. 3b). On day 4, however, 3-rank difference dominant controls showed more win frequency against subordinate KOs (statistical trend; Fig. 3c). Surprisingly, the win probability of the first two days of the test was not observed on day 4 when subordinate controls were matched against dominant KOs (Fig. 3d). We additionally analyzed the latency to win the trial for each group. We found that on both days 1 and 2, pairs of dominants from each group had increased latency compared to animals from inferior rank, and subordinate fights had the lowest latency (Fig. S4c). The same effect was observed in intermediate fights of rank 2 compared to intermediate fights of rank 3 (Fig. S4d). To assess the coping strategy for an enhanced win probability of Gαi2^−/−^ mice on the first days of the test, we measured pushing behavior. We found no differences on day 1 between groups (Fig. S5a) but found that from all victories, Gαi2^−/−^ mice presented less pushing behavior than control mice on day 2 (Fig. S5b) and no differences on day 3 (Fig. S5c). Despite this, we found no differences in the percentage of trials in which animals displayed passive (non-pushing) behavior (Fig. S5d). In general, both groups displayed more pushing behavior over the won trials on days 1 and 3 to 4 (Fig. S5e), and we found no significant differences on the percentage of total time in which animals spent pushing (Fig. S5g) although controls displayed more pushing on the first day of the test compared to the rest of the days (Fig. S5f). Overall, these results indicate that Gαi2^−/−^ mice win more frequently when confronted to non-familiar animals, but that this effect fades as successive encounters against new unfamiliars take place.

### Gαi2 ablation modulates social investigation

**Figure 4:**
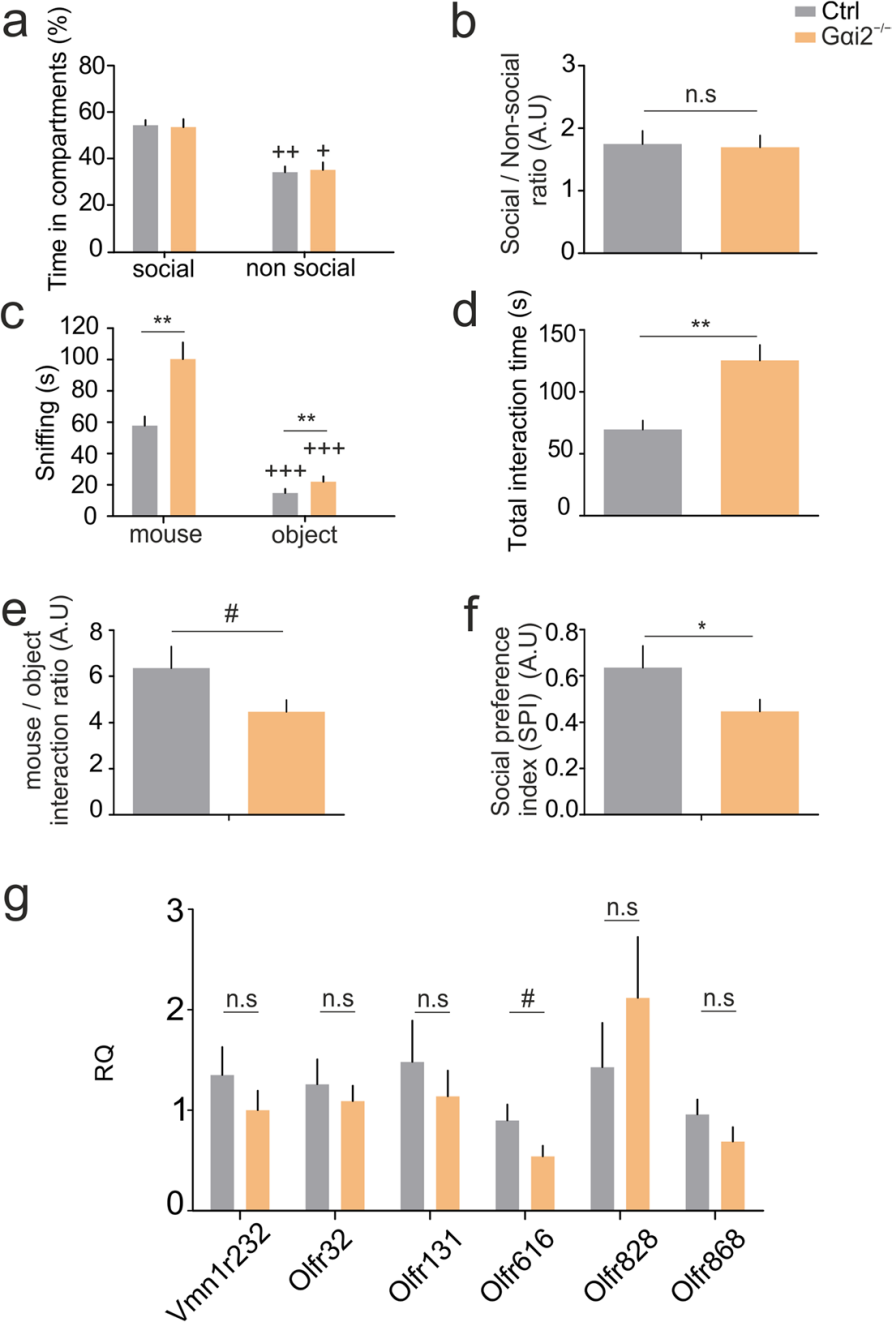
Sociability-like behavior and gene expression in mPFC of Gαi2^−/−^ mice. (**a**–**f**) Sociability, social interest, and social preference measured in the three-chamber test. (**a**,**b**) Total time and preference ratio between social (juvenile conspecific) and non-social (inert object) compartments of the 3-chamber sociability assay. Both controls (Paired T-test, p = 0.001) and Gαi2^−/−^ (Wilcoxon rank-test, p = 0.028) spent longer time in the social compartment. No differences were found between groups in the total time (**a**), neither in the social (T-test, p = 0.199) nor in the object compartment (Mann-Whitney U test, p = 0.840), n = 13 per group. No differences were found in the preference ratio (**b**) (T test, p = 0.854), n = 13 per group. (**c**,**d**) Total sniffing and total interaction times with either the mouse or the object, as a measure of social interest/investigation. Both groups, controls (Paired T-test, p < 0.0001) and KOs (Paired T-test p < 0.0001) significantly spent more time interacting with a mouse than with an object (**c**), although Gαi2^−/−^ mi**c**e spent more time than controls interacting with both items (the unfamiliar mouse (T-test, p = 0.002) and the object (T-test, p = 0.001)), n = 13 per group. Consequently, total interaction time (**d**) with both items was significantly higher in Gαi2^−/−^ mice than in controls, (T- test, p = 0.001), n = 13 per group. (**e**,**f**) Social preference measured by either interaction ratio or by the social preference index (SPI) showing a higher social preference of control animals than Gαi2^−/−^ mice. Interaction time ratio (mouse/object **e**) in control animals had a trend for increased preference compared to KOs (T-test, p = 0.090), n = 13 per group. SPI = (interaction time with the mouse minus interaction time with the object)/total interaction time (**f**), in controls had significantly increased social preference than KOs (T-test, p = 0.030). (**g**) Differential gene expression of preselected genes linked to dominance measured by real-time quantitative PCR (RT-qPCR) in medial Prefrontal Cortex (mPFC). No differences were found in the genes analyzed: Vmn1r232 (T-test, p = 0.329, n = 10 controls, n = 7 KOs), Olfr32, (Mann-Whitney U test, p = 0.725, n = 10 controls, n = 11 KOs), Olfr131 (Mann-Whitney U test, p = 0.597, n = 10 per group), Olfr616, (Mann-Whitney U test, p = 0.099, n = 8 controls, n = 11 KOs), Olfr828 (Mann-Whitney U test, p = 0.481, n = 10 controls, n = 11 KOs), Olfr868 (T-test, p = 0.221, n = 9 controls, n = 10 KOs,). All data are shown as mean ± SEM. Inter-group effects are shown as: *p < 0.05, **p < 0.01, ***p < 0.001; Within-group effects between the pre-tube test and post-tube test comparisons are shown as: ^+^p < 0.05, ^++^p < 0.01, ^+++^p < 0.001. Tendencies are represented as 0.05 > ^#^p < 0.1.

We next assessed the sociability-like behavior of Gαi2^−/−^ mice by using the 3-chamber test. We placed a wired cage containing a juvenile, unfamiliar male mouse in one of the chambers and an inanimate object in the other. Animals were allowed to freely explore the apparatus for 10 min and social behavior parameters were scored. Both mutant and control animals explored significantly more the social compartment (unfamiliar mouse) compared to the non-social one (object) (Fig. 4a). The ratio of the time spent in each compartment was not different between genotypes (Fig. 1b). These results indicate that both animals present a normal sociability-like behavior showing a clear preference for the social item. However, when we scored the investigation time, we found that Gαi2^−/−^ displayed more time investigating both the mouse and the object (Fig. 4c), and had higher total interaction times (Fig. 4d), whereas the control animals showed a trend for higher ratio between the time spent investigating the mouse and the object (Fig. 4e). Similarly, controls showed an increase in the social preference index compared to mutants (Fig. 4f). To exclude differences in activity and arousal, we calculated the total distance traveled and the activity state and found no significant differences (Fig. S7b,c,e,f). Similarly, no differences were observed in the interaction frequency between groups and genotypes (Fig. S7a,d). To measure the persistence of the social motivation of the animals, we calculated the time animals spent in each compartment in 5-minute bins. We found that control animals spent less time in the last 5 minutes and compared to KOs (Fig. S7g,h). Again, we found homogeneity within the control group between wild-type and heterozygous mice in the previous parameters mentioned (Fig. S7d,e). Together, these results indicate that Gαi2 mutants display a different social conduct, consisting of a lower interest in social investigation without altering their natural preference for a social compartment.

### Normal gene expression of markers related to dominance in a group-living social hierarchy for Gαi2-KO mutants

We have recently shown differential expression of a subset of 5 olfactory receptors and one V1R in the medial prefrontal cortex (mPFC) in group-living dominant vs. subordinate animals^20^. To test whether these genes are overexpressed in Gαi2 mutants, we performed real-time quantitative PCR in group-housed Gαi2^−/−^ mice and compared expression to group-housed controls, in both cases comprising dominant and non-dominant mice. Consistent with our previous findings, we observed robust expression of all six receptors in the mPFC of both mutant and control samples. However, we did not find any expression differences among controls and mutants for the genes analyzed, except for Olfr616 that was significantly upregulated in the controls (Fig. 4g). These results indicate that mice mutant for Gαi2^−/−^ do not display differential mPFC gene expression of olfactory markers specific for group-living dominant mice. Overall, our results suggest that group-living and first-time encounter dominance behaviors are mediated by different mechanisms.

## Discussion

**Figure 5:**
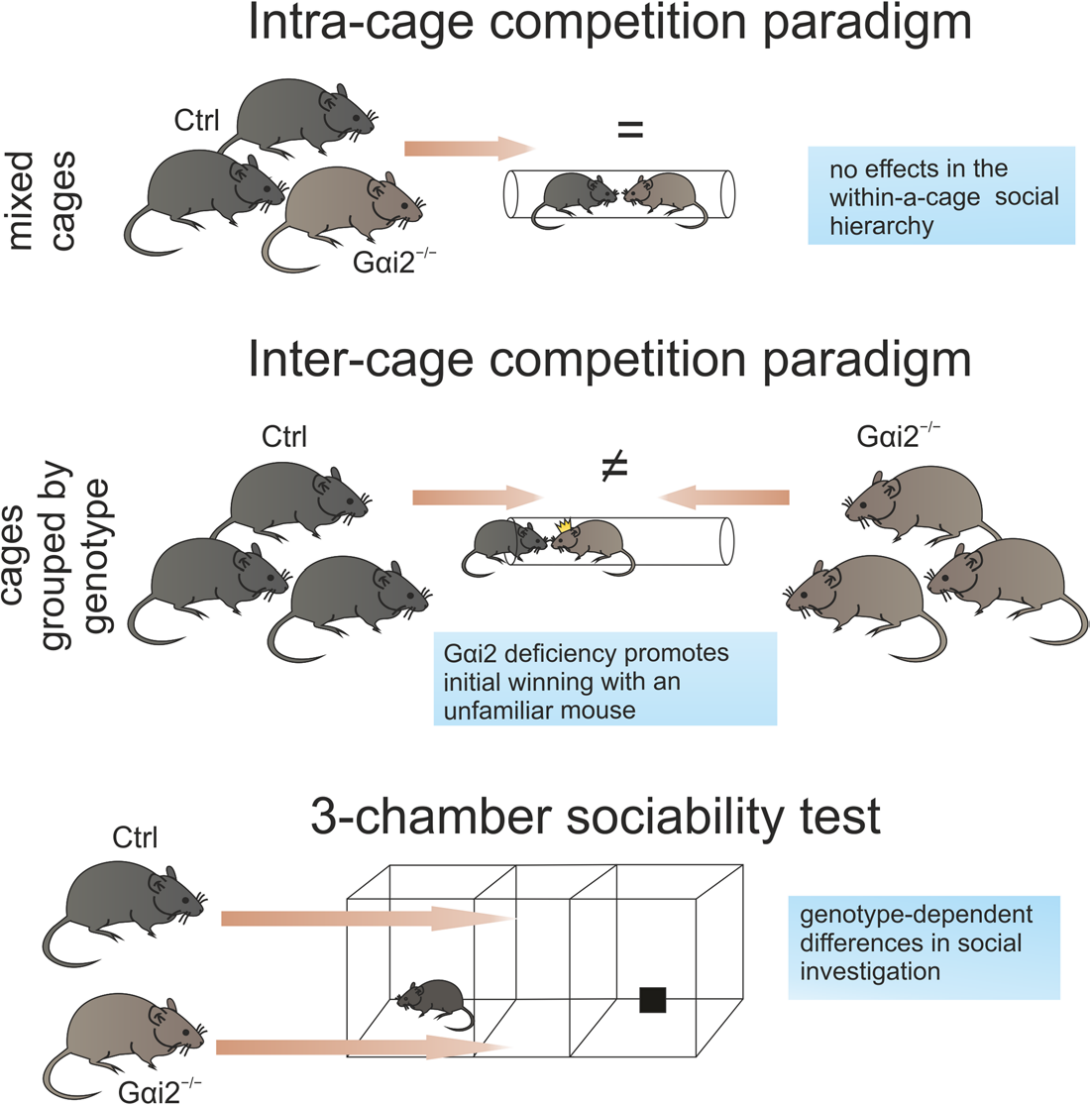
Schematic diagram summarizing the main results of our work and representing the main conclusions.

We have identified two different context-dependent forms of dominant behavior, one more related to the first stages of a social competition and another with complex within-a-cage interactions with conspecifics forming a hierarchy (Fig. 5). Specifically, we have found that pheromone signaling through the apical layer VNO receptors (such as V1R receptors) regulates the outcome of an initial social competition with an unfamiliar mouse. Conditional Gαi2-deficient mice won more encounters with control animals than randomly expected at a first-time encounter without differing at the final status allocation in a hierarchy. This supports the hypothesis that pheromone detection by the VNO has an impact on one type of social dominance but does not influence the final status acquired in a social hierarchy. These results also suggest that different circuits and behavioral processes may regulate different forms of dominance (also suggested by^14^ for the different aspects of social hierarchy, like social status recognition/perception vs. dominance behavior). Gαi2-deficient mice also displayed a lower social preference index and mouse/object interaction ratio than controls in the three-chamber test, suggesting that social investigation behavior is affected by impaired pheromone signaling in the VNO. A similar effect on social investigation was reported in vomerectomized rats^22^ (Fig. 5).

The cause for higher social dominance behavior on Gαi2-deficient mice is not due to differences in the intrinsic ability of KO mice to generate linear, stable social hierarchies, as we have found no differences in these parameters compared to controls. Stability of a hierarchy seems to be critical to induce distinct social behaviors against conspecifics, at least in rats^23^.

Deficiency in Trpc2 or Gαo in the VNO leads to reduced electrophysiological responses to pheromones and reduced aggression^17,24,25^ in the resident-intruder test. By contrast, the Gαi2-deficient mice used here were previously reported to exhibit enhanced male-male aggression^19^. Our findings suggest a new role for Gαi2, and concomitantly, for V1R neurons in the VNO, in a type of social dominance paradigm in male mice. In addition, our results suggest that aggression and dominance hierarchy are not entirely regulated by the same pathways. Conversely, aggression in the resident-intruder test may better correlate with the initial outcome of a social competition. We speculate that aggression is neither the best nor the only parameter to assess for dominance.

Finally, our results show that Gαi2-deficient mice exhibit no gene expression differences compared to controls in a given brain region and for a number of genes previously reported to be involved in group-housed animals dominance. This is consistent with our results showing lack of differences in dominance index between mutants and controls (Fig. 1). Thus, differential gene expression in group-living animals does not correlate with other forms of dominance (initial acute dominance with an unfamiliar mouse), thus supporting the hypothesis that both types of social dominance are mediated by different mechanisms. Other forms of dominance and aggression have also been reported to be mediated by distinct neural circuits (such as territorial vs infant-directed aggression^19,26^, or social status recognition vs dominance behavior^14^. In the present work, we have tried to correlate altered behavior in animals with impaired vomeronasal pheromone detection to a specific gene expression signature in the mPFC previously related to social dominance^20^. Vomeronasal function, specifically the Gαi2-expressing vomeronasal subsystem, may impact both instinctive and learned social responses that result in the establishment of hierarchies and the intensity of social interactions, mediated by interconnections between VNO epithelium, accessory olfactory bulb, medial amygdaloid complex, mPFC, and basolateral amygdala^27–29^.

Overall, our work reveals that disruption of V1R signaling cascade in the VNO promotes winning the early stages of a social competition, but it is not decisive for developing dominant positions in a social hierarchy when group-housed for several weeks. These findings unveil an interesting relationship between pheromone detection and a type of social dominance behavior, and provides support for the view that different forms of dominance may be mediated by distinct mechanisms.

## Conclusions

We report here a direct influence of pheromone detection by sensory neurons in the Gαi2–expressing layer of the VNO on territorial dominance but not on social hierarchy. This indicates that chemical communication involving these cells and their receptors is important for the outcome of an initial social competition between two unfamiliar male mice, whereas the final social status of group-housed males acquired within a given cage remains unaffected.

## Material and Methods

Animal care and experimental procedures were carried out in accordance with French and European guidelines on the protection of animals used for scientific purposes and approved by an ethical committee for animal experimentation (Comité d’Etique en Expérimentation Animale Val de Loire). Mice were kept under standard 12-h light/dark cycles with food and water ad libitum.

### Animals

Generation of the conditional Gαi2-deficient mice was done as described previously^19^. Gnai2fx/fx mice (mixed 129sv × C57BL/6 background) were crossed to mice carrying a transgene directing the expression of Cre recombinase under the control of the olfactory marker protein (Omp) promoter [Omp-Cre mice; B6; 129P2-Omptm4(cre)Mom/MomJ; Jackson Laboratory, JR 006668; backcrossed into C57BL6/J for eight generations]. Gnai2fx/fx mice carry loxP sites inserted into the introns that flank exons 2 and 4. Breeding established offspring (Gαi2^−/−^) that were homozygous for the floxed Gnai2 alleles and heterozygous for Cre and Omp (Gnai2fx/fx Ompcre/+). In these mice, Cre-mediated Gnai2 deletion was restricted to Omp-positive cells. Animals heterozygous for both alleles (Gnai2fx/+ Ompcre/+) and C57BL6/J (Janvier, France) wild-type animals served as controls. In each experiment, animals ranging from 3 to 5 months of age at the moment of testing were used.

### Experimental design

#### Intra-cage analysis of the dominance or subordinate allocations within a hierarchy of group-housed KO mice with controls

To test if Gαi2^−/−^ mice showed an increased probability of being more dominant or subordinate compared to controls during the establishment of a social hierarchy, we group-housed Gαi2^−/−^ mice together with controls. The control group consisted of Gαi2^+/−^ as well as C57BL6/J wild-type mice. The numerical distribution of animals from each group within a given cage was variable depending on the experiment. In a first experiment (A), we group-housed one Gαi2^−/−^ male with 3 controls (n = 20 mice in 5 cages). In a second experiment (B), individuals from each group (2 Gαi2^−/−^ and 2 controls) were equally distributed (n = 20). In both experiments, animals from each cage were tested in a round-robin design in the tube test to assess their social hierarchy. Final allocation from each group (genotype) was calculated. Animals from experiment B were afterwards tested in a 3-chamber sociability to assess sociability-like behavior.

#### Inter-cage analysis of the territorial behavior in a one-time social competition

22 animals, 11 Gαi2^−/−^ mice and 11 controls (Gαi2^+/−^ and C57BL6/J wild-type mice) were grouped in a genotype-dependent manner in cages of 3 to 4 individuals. Animals were initially tested in the tube test in a round-robin design to assess the hierarchy of each cage. In a second phase, we tested animal pairs from different cages and opposite groups (genotype) that had never previously interacted. This experiment was considered as experiment C. To dissect the influence of the genotype in the outcome of the competition, on the first and second days we created matches with pairs of animals that had the same social rank in their respective cages. We matched animals from different ranks in their respective cages on the following days. On day 3, animals from 1 rank difference were matched. On day 4, encounters were set with animals from the maximum rank difference (3 ranks difference except for the cages with 3 mice, in which case the difference was of 2 ranks). After these experiments, animals were perfused with saline 0.9% and brains were frozen to perform gene expression analysis.

### Behavioral testing

#### Tube test for social dominance

Animals were allowed a minimum of 2 weeks of living together before being tested in the tube test. The tube test protocol was adapted from Lindzey *et al*.^30^ and consisted of 4 days of training in which animals were trained to cross from one end of a plexiglas tube (35 cm long, 3 cm diameter) to the other without backing up. During the first two days of training, each animal was allowed to cross the tube (entrance from both sides of the tube counterbalanced) for 8 consecutive times. On the third and fourth days of the training each animal was allowed to cross the tube for 4 consecutive times (counterbalanced). After the training phase, animals were tested each day until hierarchy was considered linear and stable for a minimum of 2 days. In each day of testing, all animals within a cage were tested in dyadic interactions in a round robin design paradigm. Each pair of animals was placed at the same time at both ends of the tube allowing them to meet in the middle. The trial ended when one of the two animals retreated all four limbs from the tube. Animals that retreated were considered as losers from the trial and the others designed as winners. We created a dominance index to measure the degree of dominance calculated as the average wins per day of each individual, and we used it to confirm higher differences in dominance of each group. Hierarchical status (dominants, intermediates, and subordinates) was determined by considering animals at the top, middle, and bottom of the hierarchy of every cage respectively. Apart from victories, three other direct parameters were scored: training latency, trial latency and pushing time. Latency in the last day of training was considered an indirect measure of mobility inside the tube, and it was calculated as enter and exit time interval. Latency of the matches corresponding to the duration of each trial was scored manually. In experiment C, pushing behavior, measured as the time an animal pushed the other from the tube, was also scored as a measure of active/passive winning behavior. Pushing behavior was represented as % of time pushing respect the duration of each trial.

#### 3-chamber sociability test

A 3-chambered apparatus was used to assess social preference for a social versus a non-social compartment (30 cm width, 60 cm long). The habituation phase of the test comprised 10 minutes where the mouse was allowed to freely explore the apparatus without any stimuli in the compartments. Immediately, the animal was taken back to his cage for 1 to 2 min while a unfamiliar male mouse was placed in one compartment (social) and a neutral small object in the other (nonsocial compartment). Next, the test mouse was immediately returned to the 3-chamber apparatus and allowed to explore it for 10 minutes while being recorded. The time spent in every compartment of the apparatus was calculated using Noldus Ethovision XT software (version 11), and the time of direct investigation of every social and non-social item was manually scored. Animals from the experiment B that were previously tested in the tube test were used for this test (n = 5 Gαi2^−/−^, n = 13 controls). Additionally, 8 more Gαi2^−/−^ animals that were not previously tested in the tube test were used. To see if previous testing could influence social behavior within the Gαi2^−/−^ group, behavior was scored separately considering these two groups (previous testing versus no testing) and found no differences in the parameters analyzed except for the time exploring the object (Fig. S6).

We evaluated the interaction time with both the object and the animal, defined as the time an animal spends sniffing each stimulus. This measure was used to compare preference of direct interaction. Total interaction time, social preference index (SPI) [time exploring the mouse - time exploring the object)/total interaction time] and the ratio of interaction (time exploring the mouse/time exploring the object) were also calculated.

Four direct measures were also taken into account; 5 minute bin, distance, frequency of interaction and velocity. The 5 min bin measure consists of the time the mouse is interacting in each compartment in the first and last 5 minutes, and it is used as an indirect measure of persistence of exploration.

### Gene expression analysis

#### RNA extraction and reverse transcription assay

Total RNA extraction from medial prefrontal cortex samples and reverse transcription from RNA to cDNA was performed as described^20^.

#### Quantitative PCR reactions and analysis

Triplicate *qPCR* reactions were carried out in 10 µL total volume, using TaqMan™ Fast Advanced Master Mix (Applied Biosystems Foster City, CA) in *Quant Studio 3* real time PCR instrument (Applied Biosystems Foster City,CA). Validated primers and Taqman probes (FAM-MGB) used for Taqman gene expression assays were designed and synthesized by Applied Biosystems Foster City, CA. As an internal reference GAPDH (glyceraldehyde-3-phosphate dehydrogenase) gene was used.

The amplification conditions were predenaturation (94 °C) for 20 seconds, denaturation (94 °C) for 1 second, followed by annealing/strands extension for 20 seconds at 60 °C for 40 cycles. Data were analyzed by *Quant Studio Design and Analysis software1*.5 by threshold cycle (Ct) value of each reaction analyzed by 2−ΔΔCt method. The PCR was conducted in triplicate for each animal sample with mean value calculated.

### Stability and Linearity analysis

To assess linearity, Landau’s H index was used by means of the R software (3.5.1. version) (R Core Team), and the package “compete” developed by James P. Curley (Columbia University). Initially, the results from each match and experimental day of the tube test were converted in binary sociomatrices by assigning 0 or 1 to defeat or victory respectively. One sociomatrix was generated per cage and per day of test and afterwards introduced to the R studio (1.1.456 version). To calculate Landau’s h’, the number of randomizations was set at 10000. A single value of h’ was assigned for each sociomatrix, therefore, a single value of h’ was calculated per cage per each day of test. Average of the h’s values along the days of test was considered as a measurement of linearity for a given cage.

To further analyze not only the existent order of a cage (linearity) but also to assess if Gαi2^−/−^ animals are able to create stable hierarchies, we created two indexes: First, the Pallé-Luskey Index, (PLSI) can be applied individually (by subject) and globally (within a cage). It is based on the absolute values of variations in the rank order for each animal through time, so the individual PLSI (the rank order stability of a specific subject) is obtained by averaging the numerical values corresponding to changes in rank order, independently of a change on the subject’s hierarchical status. For instance, if a subject rank is 2^nd^ in the first day, and 4^th^ in the second day (because only consecutive days are considered), then the numerical value of the rank order change is 2. If a subject ranked 4^th^ on the second day, a change of rank to the 1^st^ position in the following day would imply now being attributed to a value of 3, corresponding to the second variation. At the end of the test, individual PLSI is considered as the average of all the rank changes along consecutive days of the test. Global PLSI (PLSI per cage) is calculated as the average of all of the individual PLSI values in a given cage. Individual and global PLSI do not have a minimum and maximum value, so they must be used as a relative indicator of stability, rather than absolute: i.e. when a subject or cage obtains a higher PLSI value than another subject or cage, it is considered that the first one has lower hierarchical stability. We then compared individual PLSI between mice of experimental groups in order to study whether there was any significant difference in individual rank order stability between subjects of different genotypes. Finally, we also compared global PLSI of Control Vs. Gαi2^−/−^ cages.

Second, the Tezanos Stability Index (TSI) was developed to study the hierarchy stability based on the dominance index (DI) of each animal. TSI ranges from 0 to 1 (low to high stability respectively) and is calculated using the variance of all DI values (each one corresponding to one animal) from the hierarchy. TSI is based on 2 assumptions: More stable hierarchies tend to have higher DI variance than less stable ones, and conversely, less stable hierarchies tend to have lower DI variance than more stable ones. When a hierarchy is fully stable, a subject tends to win all dominance encounters, and another tends to lose in all tests. Intermediate animals show victories and defeats. We can obtain scores (Sc) and the derived DI of animals from a hypothetical hierarchy with perfect stability, knowing the number of animals in the cage (n) and the number of days they perform the tube test (D). Then:

$${{\rm{Sc}}}_{{\max }}=D(n-1)$$

This allows us to obtain the score of the dominant subject in this hypothetical hierarchy, i.e. the maximum score that the dominant animal could have in a 4 subjects hierarchy which has been tested 3 days, is *Sc* = 9 (and DI = 3). To know the score of the individual in 2nd rank in this hypothetical stable hierarchy, then:

$$\begin{array}{rcl}{\rm{Sc}}(2) & = & {{\rm{Sc}}}_{{\max }}-D=9-3=6;\,{\rm{its}}\,{\rm{DI}}\,{\rm{will}}\,{\rm{be}}\,{\rm{Sc}}(2)/{\rm{D}}\\ {\rm{In}}\,{\rm{the}}\,{3}^{{\rm{rd}}}\,{\rm{rank}},\,{\rm{Sc}}(3) & = & {{\rm{Sc}}}_{{\max }}-{\bf{2}}D=9-6=3;\,{\rm{its}}\,{\rm{DI}}={\rm{Sc}}(3)/{\rm{D}}\\ {\rm{In}}\,{\rm{the}}\,{4}^{{\rm{th}}}\,{\rm{rank}},\,{\rm{Sc}}(4) & = & {{\rm{Sc}}}_{{\max }}-{\bf{3}}D=9-9=0;\,{\rm{its}}\,{\rm{DI}}={\rm{Sc}}(4)/{\rm{D}}\end{array}$$

Next, knowing the variance of the DI of all the subjects from a hypothetical hierarchy with a perfect stability H, and the variance of the DI of all the subjects from an actual and specific hierarchy J, we can calculate the TSI of the hierarchy J with this formula:

$$TS{I}_{J}=\frac{{S}^{2}[DI(J)]}{{S}^{2}[DI(H)]}$$

Then, a TSI value of a given J hierarchy can be obtained with the quotient of the DI variance from a specific hierarchy and the DI variance from a hypothetical hierarchy without animals that change in social rank along the days. The H hierarchy is constructed with the same number of animals and days of tests as the J hierarchy. However, cases of co-dominance (or co-subordination) or tied dyads can introduce some error in the interpretations, so it will help to firstly observe the scores in the socio-matrix before calculating this index. Finally, TSI indexes of Gαi2^−/−^ and Control hierarchies are compared in order to determine whether any significant difference in stability exists.

### Statistics

All data were analyzed using SPSS Statistics (v.24.0.0; IBM SPSS, Chicago, IL, USA). To test for normality, the Shapiro-Wilk test was applied. Statistically extreme cases were excluded from the analysis in every case except in those situations in which it dramatically reduced the n. ANOVA with 1 or 2 independent variables, 1- and 2-way ANOVA, respectively, were used. If data did not meet the criteria for normality and homogeneity of variances, a Kruskal-Wallis test followed by a post hoc Mann-Whitney U test was used. For comparisons between 2 independent groups, the Student’s t test was applied for normal distributions. Otherwise, the Mann-Whitney U test was used. For comparisons between 2 related groups, the paired sample Student’s t test and the Wilcoxon signed-rank test were used for normal and non-normal distributions, respectively. For proportion comparisons, Chi-square was used for independent proportions, and a binomial distribution of proportions for paired samples. The results were expressed as Mean ± SEM. In all cases, the level of significance is 0.05. Intergroup differences were expressed as * p < 0.05; ** p < 0.01; *** p < 0.001 while the intra-group differences as ^+^p < 0.05; ^++^p < 0.01; ^+++^p < 0.001. All graphs were created in Prism v.5 (GraphPad Software, La Jolla, CA, USA).

### Ethics approval

Animal care and experimental procedures were carried out in accordance with French and European guidelines on the protection of animals used for scientific purposes and approved by an ethical committee for animal experimentation (Comité d’Etique en Expérimentation Animale Val de Loire).

## Supplementary information

Supplementary Information.

## Data Availability

All materials, data and associated protocols used in this work are available to readers.

## References

[1] Wolff, J. O. & Sherman, P. W. *Rodent societies: an ecological and evolutionary perspective*. (University of Chicago Press, 2008).

[2] Laskowski Kate L., Wolf Max, Bierbach David (2016). The making of winners (and losers): how early dominance interactions determine adult social structure in a clonal fish. Proceedings of the Royal Society B: Biological Sciences.

[3] Latham N, Mason G (2004). From house mouse to mouse house: the behavioural biology of free-living Mus musculus and its implications in the laboratory. Applied Animal Behav. Science.

[4] Holy TE, Dulac C, Meister M (2000). Responses of vomeronasal neurons to natural stimuli. Science.

[5] Mohrhardt J, Nagel M, Fleck D, Ben-Shaul Y, Spehr M (2018). Signal Detection and Coding in the Accessory Olfactory System. Chem Senses.

[6] Stowers L, Kuo TH (2015). Mammalian pheromones: emerging properties and mechanisms of detection. Curr Opin Neurobiol.

[7] Chamero P (2007). Identification of protein pheromones that promote aggressive behaviour. Nature.

[8] Hausfater G, Altmann J, Altmann S (1982). Long-Term Consistency of Dominance Relations Among Female Baboons (Papio cynocephalus). Science.

[9] Rose RM, Holaday JW, Bernstein IS (1971). Plasma testosterone, dominance rank and aggressive behaviour in male rhesus monkeys. Nature.

[10] Stagkourakis S (2018). A neural network for intermale aggression to establish social hierarchy. Nat Neurosci.

[11] Syme GJ (1974). Competitive orders as measures of social dominance. Animal Behaviour.

[12] Francis RC (1984). The effects of bidirectional selection for social dominance on agonistic behavior and sex ratios in the paradise fish (Macropodus opercularis). Behaviour.

[13] Benton D, Dalrymple-Alford JC, Brain PF (1980). Comparisons of measures of dominance in the laboratory mouse. Animal Behaviour.

[14] Wang F, Kessels HW, Hu H (2014). The mouse that roared: neural mechanisms of social hierarchy. Trends Neurosci.

[15] Lindzey G, Winston H, Manosevitz M (1961). Social dominance in inbred mouse strains. Nature.

[16] Wang F (2011). Bidirectional control of social hierarchy by synaptic efficacy in medial prefrontal cortex. Science.

[17] Chamero P (2011). G protein G(alpha)o is essential for vomeronasal function and aggressive behavior in mice. Proc Natl Acad Sci USA.

[18] Zufall F (2014). TRPs in olfaction. Handb Exp Pharmacol.

[19] Trouillet AC (2019). Central role of G protein Galphai2 and Galphai2(+) vomeronasal neurons in balancing territorial and infant-directed aggression of male mice. Proc Natl Acad Sci USA.

[20] Palle A (2019). Social dominance differentially alters gene expression in the medial prefrontal cortex without affecting adult hippocampal neurogenesis or stress and anxiety-like behavior. FASEB J.

[21] So N, Franks B, Lim S, Curley JP (2015). A Social Network Approach Reveals Associations between Mouse Social Dominance and Brain Gene Expression. PLoS One.

[22] Bluthe RM, Dantzer R (1993). Role of the vomeronasal system in vasopressinergic modulation of social recognition in rats. Brain Res.

[23] Krames L, Carr WJ, Bergman B (1969). A pheromone associated with social dominance among male rats. Psychonomic Science.

[24] Leypold BG (2002). Altered sexual and social behaviors in trp2 mutant mice. Proc Natl Acad Sci USA.

[25] Stowers L, Holy TE, Meister M, Dulac C, Koentges G (2002). Loss of sex discrimination and male-male aggression in mice deficient for TRP2. Science.

[26] Isogai Y (2018). Multisensory Logic of Infant-Directed Aggression by Males. Cell.

[27] Mohedano-Moriano A (2007). Segregated pathways to the vomeronasal amygdala: differential projections from the anterior and posterior divisions of the accessory olfactory bulb. Eur J Neurosci.

[28] Pardo-Bellver C (2012). Differential efferent projections of the anterior, posteroventral, and posterodorsal subdivisions of the medial amygdala in mice. Front Neuroanat.

[29] Felix-Ortiz A.C (2016). Bidirectional modulation of anxiety-related and social behaviors by amygdala projections to the medial prefrontal cortex. Neuroscience.

[30] Isogai Y (2018). Multisensory Logic of Infant-Directed Aggression by Males. Cell.

