# Supplementary material for: Gαi2+ vomeronasal neurons govern the initial outcome of an acute social competition
Source: Sci Rep. 2020 Jan 21;10:894. doi: 10.1038/s41598-020-57765-6 (PMC6972791; doi:10.1038/s41598-020-57765-6)
Supplement: Supplementary file 1 — Supplementary Information. [file 41598_2020_57765_MOESM1_ESM.pdf]

SUPPLEMENTAL MATERIAL FOR MANUSCRIPT “Gαi2$^+$ vomeronasal neurons govern the initial outcome of an acute social competition”

Authors:
Anna Pallé[1], Marta Montero[2], Silvia Fernández[3], Patricia Tezanos[4], Juan A. de las Heras[5], Valerie Luskey[6], Lutz Birnbaumer[7], Frank Zufall[8], Pablo Chamero[9], José Luis Trejo*[10]+

SUPPLEMENTAL FIGURES

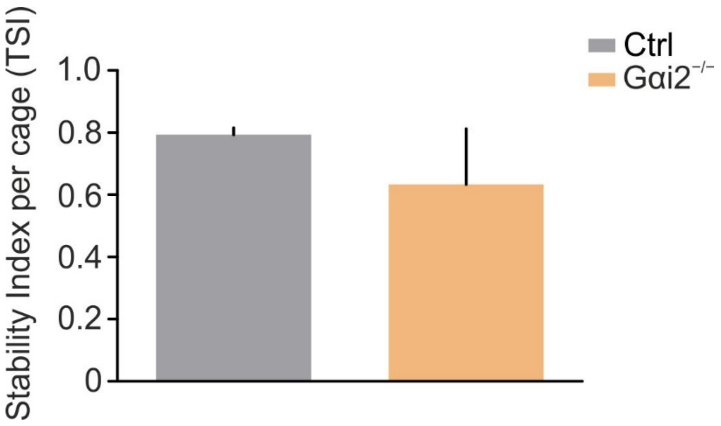

FIGURE S1

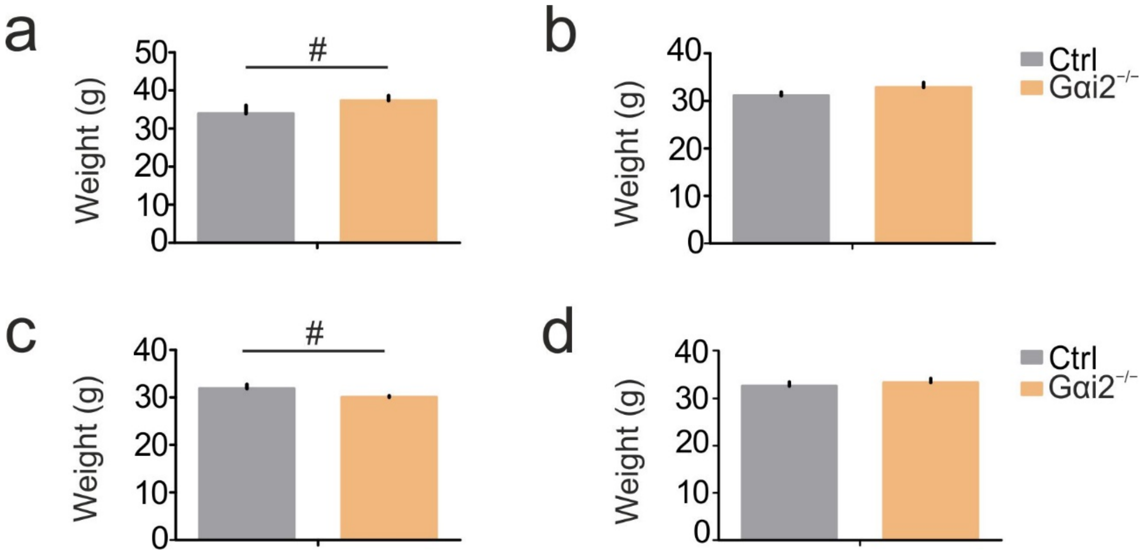

FIGURE S2

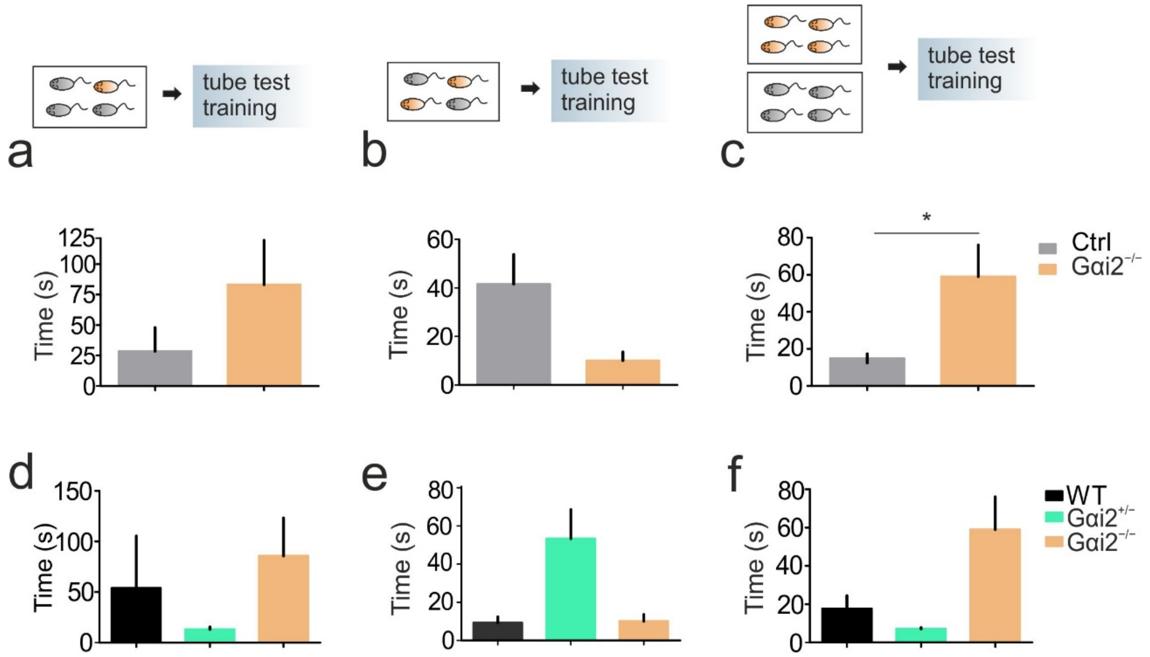

FIGURE S3

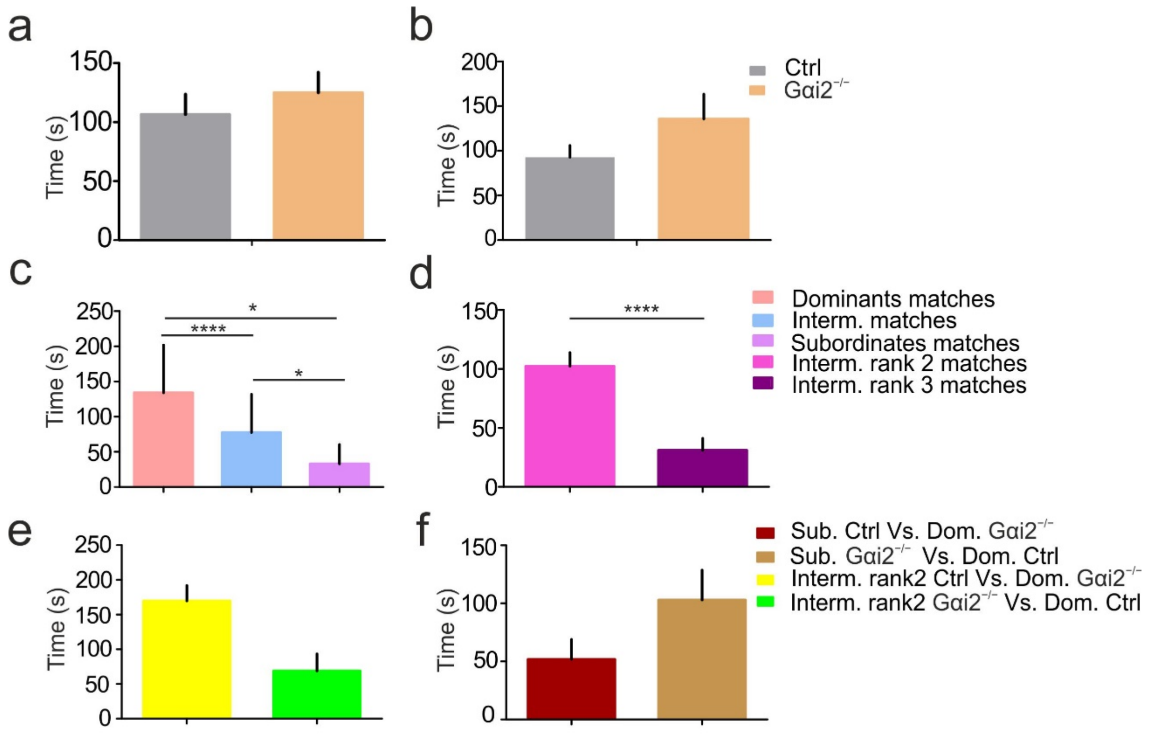

FIGURE S4

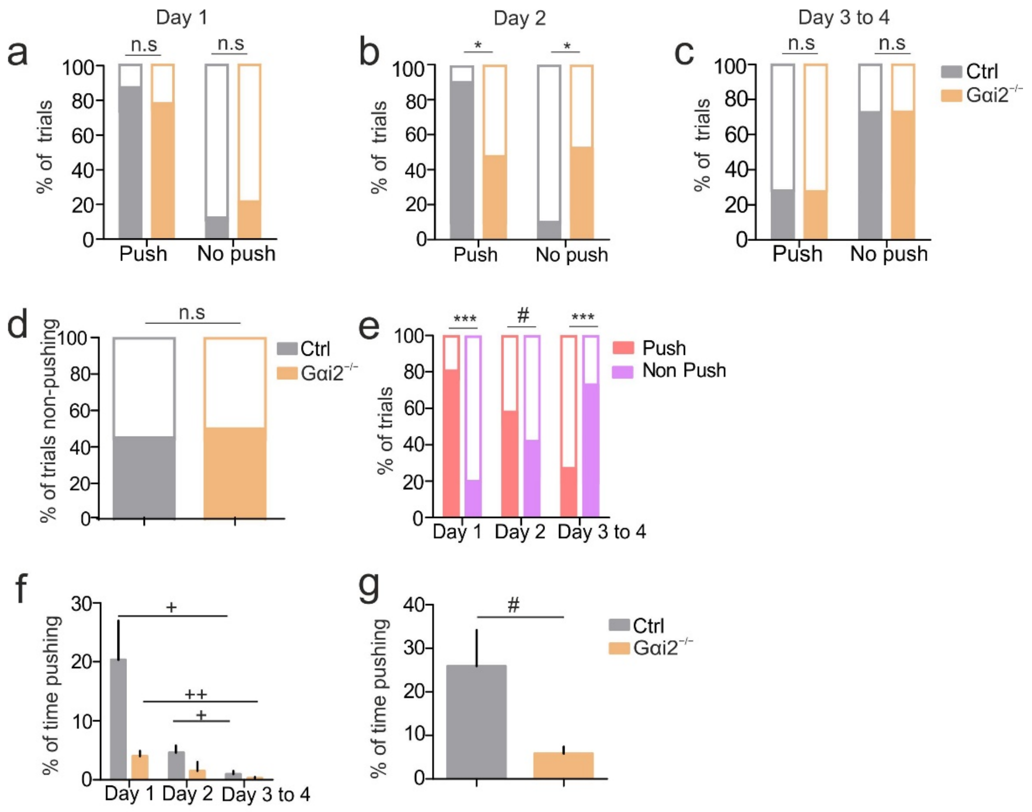

FIGURE S5

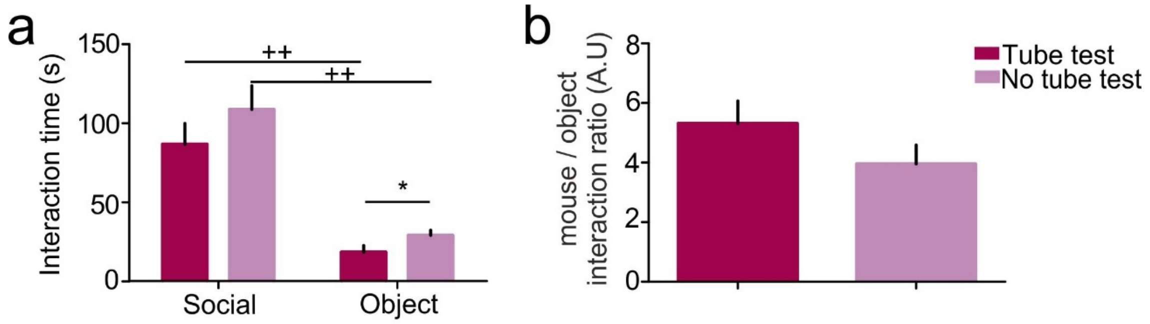

FIGURE S6

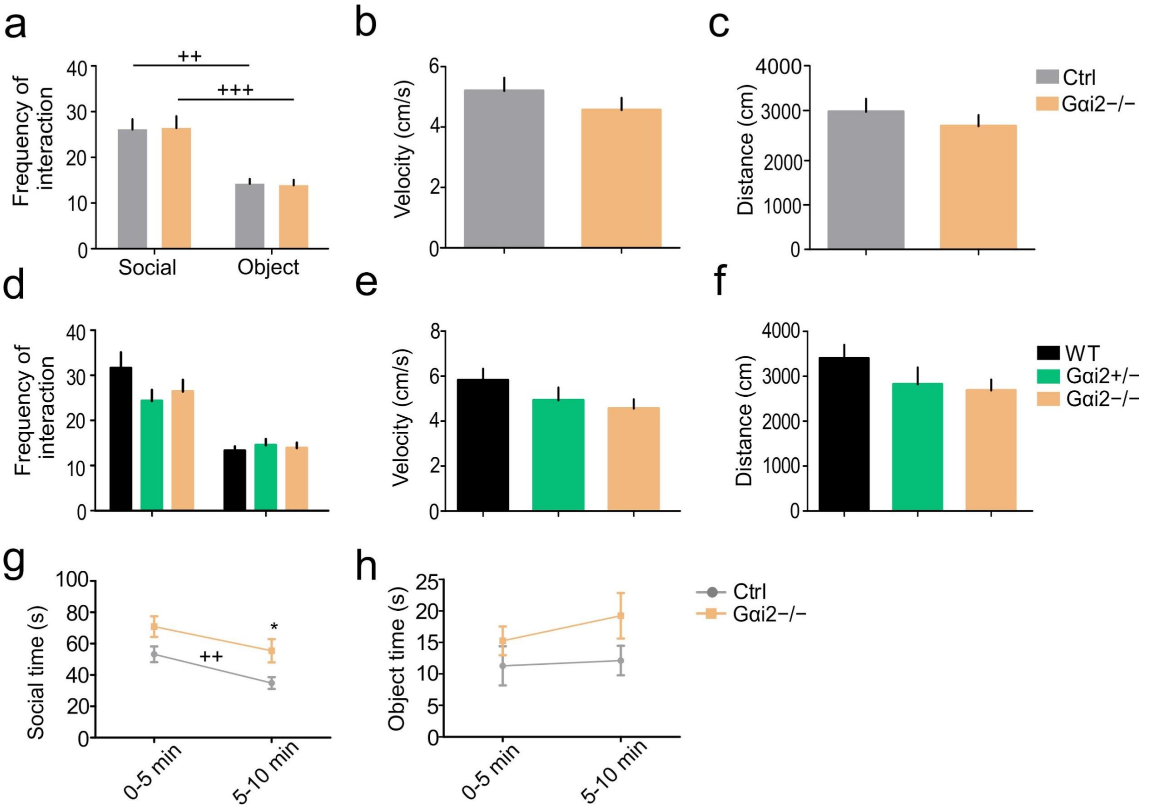

a
b
c
d
e
f
g
h
Frequency of interaction
Social
Object
++
+++
Velocity (cm/s)
Distance (cm)
Ctrl
Gαi2−/−
WT
Gαi2+/−
Social time (s)
Object time (s)
0-5 min
5-10 min
*

FIGURE S7

SUPPLEMENTAL FIGURE LEGENDS

Supplementary Figure S1. Tezanos Stability Index (TSI). TSI value for a given hierarchy (into one cage) is calculated through the quotient between the DI variance from that hierarchy and the DI variance from a hypothetical hierarchy with no variations in DI along several days. No differences were found in the stability measured by the TSI between control and Gαi2$^{-/-}$ cages, T-test, p=0.467, n= 3 cages per genotype.

Supplementary Figure S2. Body weight. a, differences between control and Gαi2$^{-/-}$ mice in the experiment with 1 KO and 3 controls per cage, Ctrl (n=10), KO (n=10), U de Mann-Whitney p=0,063. **b,** body weight differences between control and KO animals in the experiment with 2 KO and 2 controls per cage, Ctrl (n=14), KO (n=5), U de Mann-Whitney p=0,130**. c,** differences between control and KO animals in the first-time encounters experiment. Ctrl (n=11), KO (n=11), T-student p=0,057**. d,** body weight differences between control and ko animals (considering the 3 experiments together), Ctrl (n=34), KO (n=26), U de Mann-Whitney p=0,146.

Supplementary Figure 3. Latency to cross the tube during training. a-c, Differences in training latency between control and ko animals; **a**, experiment with 1 KO and 3 controls per cage, Ctrl (n= 9), KO (n=10), U de Mann Whitney p= 0,182. **b**, experiment with 2 KO and 2 controls per cage, Ctrl (n= 15), KO (n=4), U de Mann Whitney p= 0,1. **c**, first-time encounters experiment, Ctrl (n=11), KO (n=11), T-Student p=0,022. **d-f**, Differences in training latency per genotype; **d**, experiment with 1 KO and 3 controls per cage, Ctrl (n=4), Heterozygous (n=6), KO (n=10), Kruskal-Wallis p=0,308. **e**, experiment with 2 KO and 2 controls per cage, Ctrl (n=4); Heterozygous (n=11), KO (n=4), Kruskal-Wallis p=0,128. **f**, first-time encounters experiment, Ctrl (n=8), Heterozygous (n=3), KO (n=11), Kruskal-Wallis p=0,302.

Supplementary Figure 4. Latency to win a trial in the different experiments. a-b, latency to win the trial in **a**, experiment with 1 KO and 3 controls per cage, Ctrl (n= 10), KO (n=10), U de Mann Whitney p=0,542, and **b**, experiment with 2 KO and 2 KO per cage, Ctrl (n= 15), KO (n=5), U de Mann Whitney p=0,544. **c-f**, first-time encounters experiment; **c**, latency to win a trial between animals of the same rank:

Dom vs Dom (n =18), Sub vs Sub (n=18), Int vs Int (n=26). Kruskal-Wallis for independent samples: Sub vs Sub – Int vs Int p= 0,026; Sub vs Sub – Dom vs Dom p< 0,0001; Dom vs Dom – Int vs Int p= 0,043. **d**, latency to win a trial between intermediate animals of the same rank: Int2 vs Int 2 (n= 17), Int3 vs Int3 (n=9), U de Mann-Withney p<0,0001. **e-f**, latency to win a trial between animals of different rank; **e**, 1 position rank difference, Int2 WT vs Dom KO (n=8), INT2 KO vs Dom WT (n=9), Kruskal-Wallis p=0,277. Int3 WT vs Sub KO (n=6), INT3 KO vs Sub WT (n=6), Kruskal-Wallis p=0,467). **f**, 3 positions rank difference, Sub WT vs Dom KO (n=8), sub KO vs Dom WT (n=9), Kruskal Wallis p=0,336.

Supplementary Figure 5. Active versus passive winning behavior in the inter-cage tests (only won trials). a-c, percentage of trials with active (pushing) or passive (non-pushing) behavior; **a**, first-day matches. No differences were found between Gαi2$^{-/-}$ mice and controls. Squared-Chi, Chi value 0.325, p=0.569. Ctrl n=8 trials, Gαi2$^{-/-}$ n= 23 trials. **b**, second-day matches. Controls displayed a higher percentage than Gαi2$^{-/-}$ mice. Squared-chi, Chi value 4.188, p=0.041. Ctrl n=10 trials, Gαi2$^{-/-}$ n= 21 trials. **c**, days 3 and 4. No differences were found between Gαi2$^{-/-}$ mice and controls. Squared-Chi, Chi value 0.001, p=0.978. Ctrl n=22 trials, Gαi2$^{-/-}$ n=26 trials. **d**, percentage of winning trials with passive (non-pushing) behavior. No differences were found between groups. Squared chi, Chi value 0.255, p=0.614. Ctrl n=40 trials, Gαi2$^{-/-}$ n=70 trials. **e**, percentage of trials with active (pushing) or passive (non-pushing) behavior per day. On day 1 (Binomial distribution, p= 0.00034) animals differentially displayed more pushing behavior in contrast with days 3 to 4 (Binomial distribution, p= 0.00069) when animals displayed more passive (non pushing) behavior of winning. A trend was observed on day 2 (Binomial distribution, p= 0.09604). **f**, Average percentage of time spent pushing during the trial per day. Ctrl day1 (n= 8), KO day1 (n=23), Ctrl day2 (n=9), KO day2 (n=22), Ctrl day3 (n=22), KO day3 (n=26). Kruskall-Wallis → KO day3 - Ctrl day3 p=1.000, KO day1 – Ctrl day1 p=1.000, KO day2 – Ctrl day2 p=0.286, KO day3 – KO day2 p=1.000, KO day3 – KO day1 p=0.001, KO day2 – KO day1 p=0.2719, Ctrl day3 – Ctrl day2 p=0.024, Ctrl day3 – Ctrl day1 p=0.016, Ctrl day2 – Ctrl day1 p=1.000. **g**, Average percentage of time spent pushing during the trial per group. Ctrl n=36, KO n=60. U de Mann-Whitney p=0.064.

Supplementary Figure 6. Differences in the three-chamber sniffing behavior between Gαi2$^{-/-}$ mice as a result of tube test performance. a, differences in interaction time between tube tested and not tested animals per compartment. KO tube test n=5, KO no tube test n=8. T-Student independent samples (intergroup): mouse p=0,336, object p=0,069. T-Student paired samples (intragroup): Tube test p=0,003, no tube test p=0,001. **b**, differences in mouse/object interaction time ratio. KO tube test n=5, no tube test n=8. T-Student independent samples p=0,201.

Supplementary Figure 7. Three-chamber analyses and breakdown for genotype. a, differences in interaction time frequency per compartment. Ctrl n=12, KO n=11. T-Student independent samples (intergroup): mouse p=0,802, object p=0,793. T-Student paired samples (intragroup): Ctrl p=0,001, KO p<0,0001. **b**, differences in velocity. Ctrl n=13, KO n=13. T-Student independent samples p=0,284. **c**, differences in distance traveled into the chamber. Ctrl n=13, KO n=12. T-Student independent samples p=0,132. **d**, differences in interaction time frequency per compartment. WT n=13, Heterozygous n=3, KO n=9. Repeated measures ANOVA p=0,619. **e**, differences in velocity per genotype. WT n=13, Heterozygous n=4, KO n=8. One-factor ANOVA p=0,148. **f**, differences in distance traveled into the chamber per genotype. WT n=12, Heterozygous n=4, KO n=8. One-factor ANOVA p=0,24. **g,** 5-min bit breakdown of interaction time into the social compartment. Ctrl n=12, KO n=13. T-Student, independent samples (intergroup): 0-5 min p=0,228, 5-10 min p=0,025. T-Student, paired samples (intragroup): Ctrl P=0,005, KO p=0,328. **h**, 5-min bit breakdown of interaction time into the object compartment. Ctrl n=13, KO n=13. Mann-Whitney U test, (intergroup): 0-5 min p=0,466, 5-10 min p=0,142. Wilcoxon Test (intragroup): Ctrl p=0,309, KO p=0,201.
